# Collagen/Beta-Tricalcium Phosphate Based Synthetic Bone Grafts via Dehydrothermal Processing

**DOI:** 10.1155/2015/576532

**Published:** 2015-10-04

**Authors:** Burcu Sarikaya, Halil Murat Aydin

**Affiliations:** ^1^Institute of Science, Bioengineering Division, Hacettepe University, 06800 Ankara, Turkey; ^2^Faculty of Engineering and Architecture, Genetics and Bioengineering Department, Kastamonu University, 37150 Kastamonu, Turkey; ^3^BMT Calsis Health Technologies Co., 06980 Ankara, Turkey; ^4^Environmental Engineering Department & Bioengineering Division and Centre for Bioengineering, Hacettepe University, 06800 Ankara, Turkey

## Abstract

Millions of patients worldwide remain inadequately treated for bone defects related to factors such as disease or trauma. The drawbacks of metallic implant and autograft/allograft use have steered therapeutic approaches towards tissue engineering solutions involving tissue regeneration scaffolds. This study proposes a composite scaffold with properties tailored to address the macro- and microenvironmental conditions deemed necessary for successful regeneration of bone in defect areas. The biodegradable scaffold composed of porous beta-tricalcium phosphate particles and collagen type I fibers is prepared from a mixture of collagen type-I and *β*-tricalcium phosphate (*β*-TCP) particles via lyophilization, followed by dehydrothermal (DHT) processing. The effects of both sterilization via gamma radiation and the use of DHT processing to achieve cross-linking were investigated. The impact of the chosen fabrication methods on scaffold microstructure and *β*-TCP particle-collagen fiber combinations were analyzed using X-ray diffractometry (XRD), scanning electron microscopy (SEM), Fourier transform infrared spectroscopy (FTIR), differential scanning calorimetry (DSC), and microcomputerized tomography (*µ*-CT). Electron spinning resonance (ESR) analysis was used to investigate free radicals formation following sterilization. Results revealed that the highly porous (65% porosity at an average of 100 *µ*m pore size), mechanically adequate, and biocompatible scaffolds can be utilized for bone defect repairs.

## 1. Introduction

Accidents, fractures, osteoporosis, tumor removal, and hereditary diseases where large bone defects may occur necessitate appropriate methods for the regeneration of lost tissues [1]. Many clinical approaches to bone repair and regeneration have been reported. Autografts use, the current gold standard for treating critical-sized bone defects, is limited by factors such as donor site morbidity, the risk of bone resorption, and poor availability and there is thus growing interest in bone graft substitutes [2, 3]. The dry weight of the natural bone matrix consists of 75% inorganic material, calcium phosphate, and around 22% organic material, collagen along with low amounts of proteoglycans, peptides, and lipids [4]. The extracellular matrix, which is composed of a network of collagen fibrils offering an environment for cell proliferation and migration, can be decellularized. However, the risk of pathogen or disease transfer has led, instead, to the development of scaffolds that can mimic the ECM in* in vitro* conditions [5]. Scaffolds that can be used for bone regeneration are fascinating as they have the ability to be produced with desirable properties, can be sterilized to display low immunogenicity, shaped to the defect site, optimized, and altered accordingly, and can be used in combination with cells and growth factors [1]. Materials used in bone grafts must allow for osteoblasts to interact with other osteoblasts, proliferate, and migrate [6].

Materials used for scaffolds must be biocompatible, biodegradable, mechanically strong, and osteointegrative. Ceramic materials are commonly used in clinical applications; hydroxyapatite (HA) is one of the most widely used ceramics in medicine. Even though HA displays high mechanical strength, it does not possess interconnected porosity, a feature essential to the vascularization of newly formed tissue. Furthermore, as HA is nonbiodegradable it cannot be replaced with new bone tissue over time [7]. To avoid such limitations we have preferred the incorporation of highly pure and porous *β*-TCP particles (SupraBone (BMT Calsis Co., Turkey)) in the scaffolds developed in our study. *β*-TCP has a Ca/P ratio very similar to that of natural bone tissue, is biodegradable, and can be produced to exhibit ideal pore sizes [8]. However, this material cannot be used alone in the regeneration of bone due to its brittleness. For this reason it is more effective to use TCP in conjunction with the primary components of bone such as collagen. The use of collagen-only scaffolds is also not desirable due to its early degradation within the body and low resistance [9]. Furthermore, there are difficulties associated with the combination of collagen with other biopolymers as internal shear stresses between the materials tend to compromise the mechanical integrity of the scaffold [8]. Hybrid scaffolds that unite the elasticity of collagen and the high mechanical resistance of *β*-TCP offer viable solutions to the current problems encountered in hard tissue engineering [6, 10].

Many methods can be employed to create pore of desired size, density, and interconnectivity in tissue engineering scaffolds [11]. Chemical approaches, where the removal of solvents involves arduous processing, carry a risk of causing tissue damage if residue remains within the material [12]; we have thus utilized a lyophilization technique in our study to achieve porosity. As the *β*-TCP and collagen composite scaffolds are likely to degrade/disintegrate before new tissue is formed, they need to be cross-linked for* in vivo* use. Agents such as diphenylphosphoryl azide (DPPA) and glutaraldehyde (GTA) have previously been used to cross-link collagen [13]. Even though the cross-linking agents are able to give fast and effective results, the complete removal of solvents used in the process is not always successful. To achieve cross-linking whilst avoiding the possibility of such adverse effects, a nonchemical method, namely, dehydrothermal (DHT) processing, can be used. As strong links between the ceramic phase and organic phase of the collagen/*β*-TCP scaffolds can be formed via DHT processing, degradation time is increased, cell proliferation is increased, and controlled release of growth factors becomes possible [3].

In this work, porous collagen/*β*-TCP composites were prepared by acid-treating the collagen, dispersing fine *β*-TCP particles throughout the mixture, and lyophilizing in order to obtain desired uniform microenvironments within the composites. To minimize early degradation of the scaffold in the body, DHT processing to cross-link the collagen/*β*-TCP scaffold was used. The effects of cross-linking and sterilization parameters on *β*-TCP particles and collagen properties were investigated.

## 2. Materials and Methods

### 2.1. Preparation of Porous *β*-TCP/Collagen Scaffolds

Collagen type-I (RUP, Czech Republic) was dispersed in a diluted acetic acid solution (0.1 N) at room temperature. To completely dissolve collagen, the mixture was stirred for 5 minutes using an overhead blender at the speed of 500 rpm. In order to deliver the maximum amount of ceramic particles without compromising the handling and wettability properties of the final graft material; an 80 : 20 w/w ratio was selected for *β*-TCP/collagen mixture. A designed amount of *β*-TCP powders was slowly added into the collagen suspension by stirring. *β*-TCP (SupraBone, 0,5–1 mm particle size) was kindly donated by BMT Calsis Health Technologies, Co., Turkey. This particle size was found suitable since bigger ceramic particles could lead to lower packing density and fragile structure. After the addition of *β*-TCP, the slurry mixture was stirred for 3 minutes at a speed of 250 rpm. The slurry was casted into a Teflon mold (dimensions 5 mm × 25 mm × 100 mm) and stored at −80°C for 3 hours [14]. Subsequently, the frozen mixture was lyophilized to obtain a porous collagen/*β*-TCP composite. After freeze-drying the porous composites, the matrices were placed into the vacuum oven for dehydrothermal processing at 3 mbar and 110°C operating conditions for 1, 3, and 5 days. It should be noted that the shorter treatment times may lead to incomplete cross-linking while longer periods could effect the chemical structure. Some of the bone grafts were sterilized by using gamma irradiation with an average prescribed dose of 25 kGy at room temperature, whilst a control batch remained unsterilized for comparative analysis purposes.

### 2.2. Characterization of Scaffolds

The crystalline phases of the porous composites were determined with an X-ray diffractometer (XRD) using the RIGAKU, RINT 2000 system. The surface morphology of *β*-TCP/collagen scaffolds was examined using a scanning electron microscope (SUPRA 50VP, Germany). The scaffolds were sputter-coated with gold-palladium alloy before SEM imaging. The operated voltage was set at 20 kV. FTIR analysis was carried out to determine components of *β*-TCP/collagen scaffolds and characterize the interactions between *β*-TCP particles and collagen fibrils using an attenuated total reflection system on a (BRUKER TENSOR 27, USA) spectrometer. Further analysis involving Fourier transform infrared (FTIR) spectroscopy was adopted using the KBr pellet method and FTIR spectra were recorded at 4.0 cm^−1^ resolution. A dry system was used to prevent atmospheric moisture. Differential thermal analyses (DTA) were performed for the determination of thermal transitions. The thermal behaviors of the *β*-TCP/collagen scaffolds were also investigated by thermogravimetric analysis (TGA), and differential thermal analysis (DTA) using the NETZSCH thermal analyzer (STA 449 F3 JUPITER, Germany). For TG-DTA measurement, the heating rate was set at 10°C/min under flowing air. An X-band Electron Spin Resonance (ESR) spectrometer (Bruker ELEXSYS E580, USA), fitted with a TE_102_ cavity and operating at 9.85 GHz microwave frequency and 100 kHz magnetic-field modulation frequency, was used to test all samples in this study.

In order to reveal mechanical properties, three-point bending strength tests were carried out using a Zwick/Roell Z250 mechanical tester. The size of the specimens was ~5 mm × 12,5 mm × 50 mm. The 0,2 N load was applied over a 10 mm span and at the midpoint of the 12,5 mm × 50 mm surface. All tests were performed using a crosshead speed of 2 mm/min. The compression strength of porous scaffolds was also measured using a Zwick/Roell Z010 mechanical tester at a crosshead speed of 2 mm/min. The samples were rectangular in shape, with dimensions 5 mm in height and 10 mm × 10 mm in cross section. During compression test, the 0,25 N load was applied until densification of the porous samples started to occur.

A micro-CT system (SkyScan 1172, USA) was used to quantify the 3D microstructural properties of the collagen/*β*-TCP scaffolds. Isotropic slice data were obtained by the system and reconstructed into two-dimensional images and analyzed to produce 3D images for quantitative architectural parameters. The data were further analyzed by the software CT Analyzer (SkyScan, USA). Porosity, pore sizes, and pore distribution were measured from the constructed 3D model.

To evaluate the degree of water uptake, the cross-linked scaffold samples with the specific dimensions (10 mm × 12 mm × 5 mm) were accurately weighed (*W*_0_) and immersed in 3 mL PBS solution at 37°C according to [15]. The samples were reweighed at regular time intervals (*W*_*S*_) to find the equilibrium swelling. The water uptake was defined as (*W*_*S*_ − *W*_0_) × 100/*W*_*S*_.

### 2.3. Cell Culture Assays

The scaffolds consisting of collagen and *β*-tricalcium phosphate (TCP) were cut to size of 24 mm × 47 mm prior to the commencement of the experiment. To assess the possible cytotoxicity of the scaffolds, they were seeded with a commonly used osteosarcoma cell line, MG63. The cells were exclusively cultured for this experiment using a specifically supplemented medium consisting of *α*-MEM, 10% FBS, 1% L-glutamine, 1% antibiotics, and antimycotic. Prior to seeding, scaffolds were cut to a specific size (6 mm × 6 mm) and then soaked in fully supplemented MG63 medium for 30 min. To assess initial seeding efficiency, the scaffolds were then seeded in a sterile Petri dish. 0.5 × 10^6^ cells were seeded in each scaffold in a volume of 100 *μ*L supplemented MG63 medium. The cell suspension was seeded with 5 separate volumes of 20 *μ*L to ensure a homogenous distribution within the scaffold. It was also hoped that this method of seeding would prevent cell suspension leaking from the scaffolds until cell attachment was achieved. The scaffolds were then incubated (37°C at 5% CO_2_) for 2 hrs before being transferred to individual wells of a 24-well plate. Once transferred, the wells were topped up with 1 mL supplemented MG63 medium. After 24 hrs, half the wells were topped up with an additional 1 mL supplemented MG63 medium (bringing the volume of half the wells to 2 mL). This was intended to assess if medium volume affected cytotoxicity and scaffold integrity. The scaffolds were imaged at 2 hrs, 24 hrs, 48 hrs, 72 hrs, and 192 hrs. The pH of the supplemented MG63 medium was tested prior to seeding and again after the experiment was complete.

For the disintegration experiment, eight scaffolds (cut to the same dimensions as those used in the seeding experiment) were used to assess and monitor the levels of disintegration within a number of mediums: DMEM, *α*MEM, supplemented MG63 media, and PBS. The scaffolds were cut and immediately transferred to individual wells of a 24-well plate. Each well was then topped up with their designated medium (3x scaffolds were each soaked in DMEM, *α*MEM, supplemented MG63 media, and PBS). The scaffolds were imaged every 30 min for the first 5 hrs and twice again at 24 hrs. Immediately following the initial imaging at 24 hrs, an attempt was made to transfer the scaffolds to new wells with images being taken again immediately after. This was intended to evaluate whether the moving of the scaffolds after 2 hrs of postseeding could have affected the integrity of the scaffolds. MTT analysis was carried out to evaluate cell viability at 45 hrs and 70 hrs (*n* = 4 × 3) using manufacturers protocols. A standard curve was made using the same methods as those previously mentioned with cell numbers of 0.2 × 10^6^ to 1 × 10^6^.

## 3. Results

### 3.1. Scaffold Characterization

**Figure 1:**
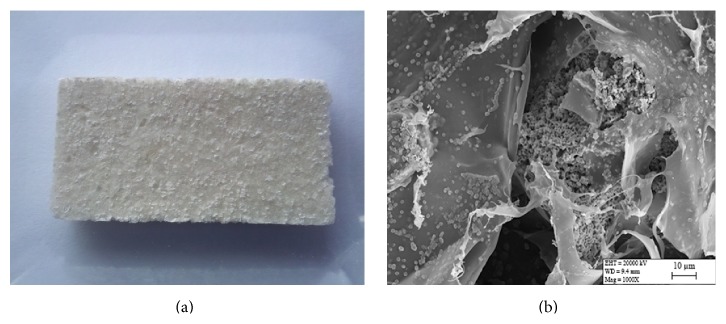
Characteristics of the collagen/*β*-TCP scaffolds. (a) Macroscopic image, (b) SEM image of gamma irradiation sterilized scaffold (×1000).

Figure 1 shows the morphology and structure of collagen/*β*-TCP scaffolds. The composite scaffolds were white and opaque with a spongy appearance (Figure 1(a)) and exhibited an interconnected porous structure (Figure 1(b)). *β*-TCP particles were distributed well and homogeneously in the skeleton network of collagen fibrils. The interconnected porous scaffold structures have beneficial effects on cell proliferation, migration, and nutritive transportation in bone tissue engineering. It was also noted that the organic and inorganic phases were combined appropriately following gamma sterilization.

**Figure 2:**
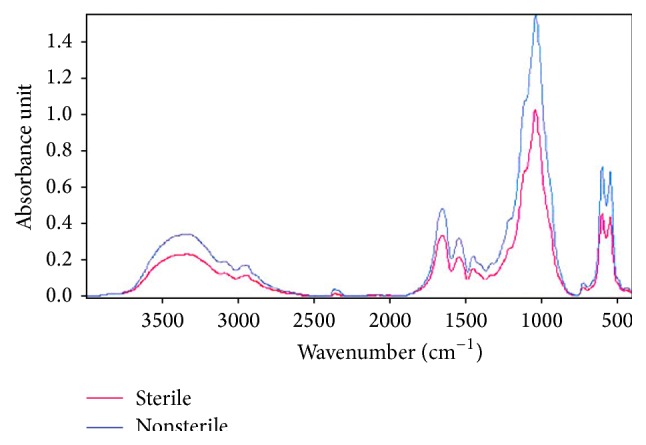
FTIR spectra of the sterile and nonsterile collagen/*β*-TCP scaffolds.

In the FTIR spectra (Figure 2), adsorption bands were clearly detected, which corresponded to *β*-TCP (PO_4_^3−^: 1000, 600, and 580 cm^−1^) and collagen (COO^−^: 1630, 1543, and 1452 cm^−1^), respectively. The FTIR data shows the collagen and ceramic phases have not undergone significant change following poststerilization.

**Figure 3:**
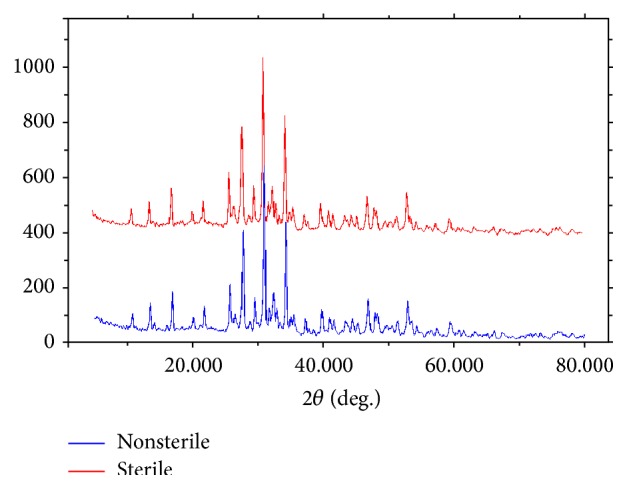
XRD patterns of collagen/*β*-TCP scaffold.

XRD investigations detected a peak profile of collagen/*β*-TCP scaffold overlapped with the product of *β*-TCP powder. Mineralogical analysis results examined in sterile and nonsterile samples were found to contain [Ca_3_(PO_4_)_2_] phase. Collagen type-I did not peak in XRD analysis due to its amorphous structure but peak between 25 and 35° in sterile and nonsterile samples revealed the presence of amorphous structure. There are not any differences observed between sterile and nonsterile samples peak when graphics overlapped (Figure 3).

Following thermal analyses, a three-stage degradation was observed in TG-DTA analysis that determines the weight loss. The first endothermic peak in thermogram that related with weight loss indicates evaporation of water. Second exothermic peak in the range of 200–400°C implies collagen degradation. A noticeable difference between the DTA results of sterile and nonsterile samples was not seen. Sterile and nonsterile samples exhibit similar thermal behavior (Table 1).

There is no ESR signal obtained from irradiated scaffolds. In the absence of fluctuation in 3500-Gauss magnetic field of free radical formation, we concluded that free radical formation was not observed in scaffolds after gamma sterilization at 25 kGy dose irradiation.

In order to see the effects of DHT processing times on the mechanical properties, compression tests were performed. The summary of the mechanical properties is given in Table 2. It was noted that a DHT processing time of 3 days is optimal, since after this point the compression modulus of the samples showed declination. In addition to the compression tests, a three-point bending test was also performed. According to three-point bending tests, Young's modulus and strength values for the sterile samples were found as 14.12 ± 0,7 MPa and 0.77 ± 0,03 MPa (*n* = 3), respectively.

**Figure 4:**
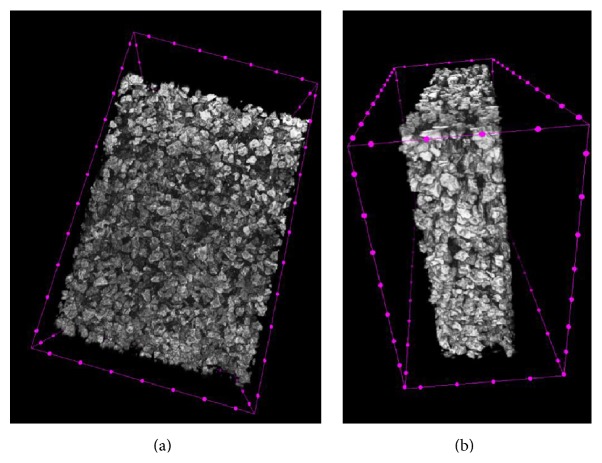
Micro-CT images of a *β*-TCP/collagen scaffold. (a) Top-view of scaffold. (b) Side-view of scaffold.

Detailed analysis results of micro-CT images are given in Table 3. The representative micro-CT images of the scaffolds were shown in Figure 4. Scaffolds have an open and porous architecture with porosity of around 65,63% in the dry stage. It was found that the ceramic phase integrated into the matrix had a micro- and nanopore structure and this had an effect on these results.

**Figure 5:**
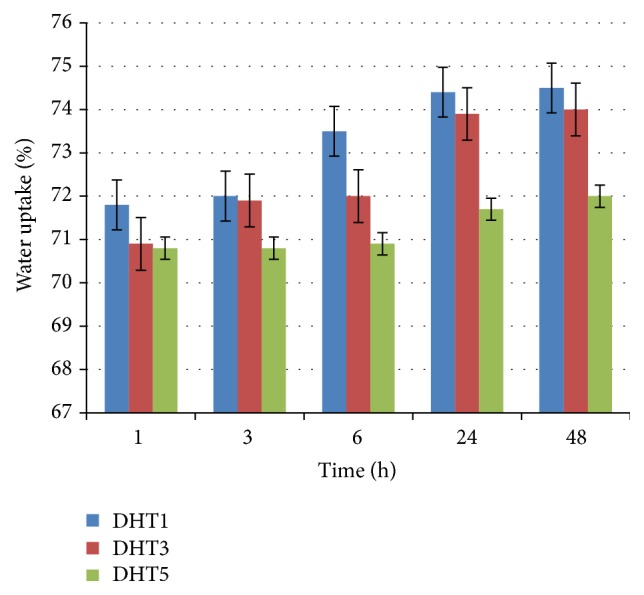
Water uptake of the *β*-TCP/collagen scaffolds after 48 hours. Error bars show means ± standard deviation for *n* = 3.

The results of the water uptake experiment performed for the cross-linked scaffolds revealed that all the scaffolds reached the equilibrium swelling point in less than 48 hours. As observed in Figure 5, water content of the scaffolds was inversely proportional to the DHT processing time (DHT1, DHT3, and DHT5 denote 1, 3, and 5 days of DHT treatment).

### 3.2. *In Vitro* Disintegration Tests

**Figure 6:**
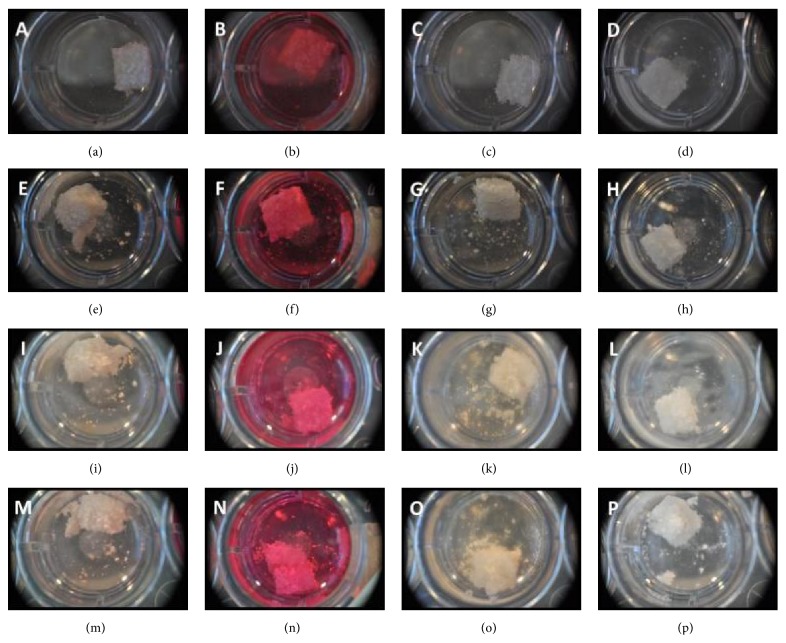
Morphology of scaffolds soaked in four different mediums. (a)–(d) 1 hr after soak. (e)–(h) 5 hrs after soak. (i)–(l) 24 hours after soak. (m)–(p) After transfer (24 hours). (a), (e), (i), (m) Alpha-MEM. (b), (f), (j), (n) DMEM. (c), (g), (k), (o) MG63 supplemented medium. (d), (h), (l), (p) PBS.

Disintegration was evident in all four mediums within just 1 hr of being immersed. Within 5 hours, the disintegration had reached a point where the scaffolds had begun to disintegrate (Figure 6). After 24 hrs, the scaffolds had become so disintegrated that they could not be transferred to another well. The scaffolds had become too soft and could not be handled.

**Figure 7:**
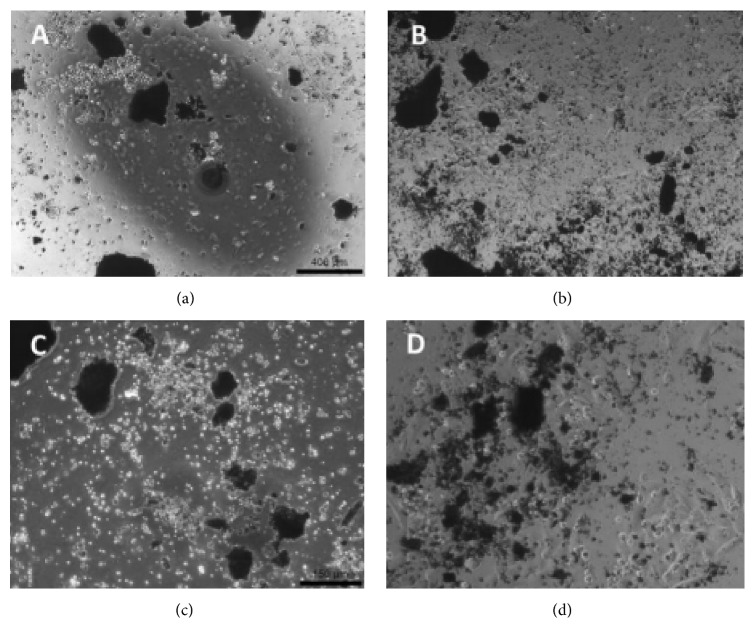
Images of the initial seeding of the scaffolds. (a) and (c) 2 hrs after seeding. (b) and (d) 24 hrs after seeding. (a) and (b) ×4 magnification. (c) and (d) ×10 magnification.

The initial seeding of the scaffolds was considered successful given that each scaffold accepted the supplemented MG63 medium (30 min soaking) and cellular solution without leaking. It was noted when soaking and seeding the scaffolds that when more time was given to the scaffolds to accept a solution, they retained the solution better (Figure 7).

**Figure 8:**
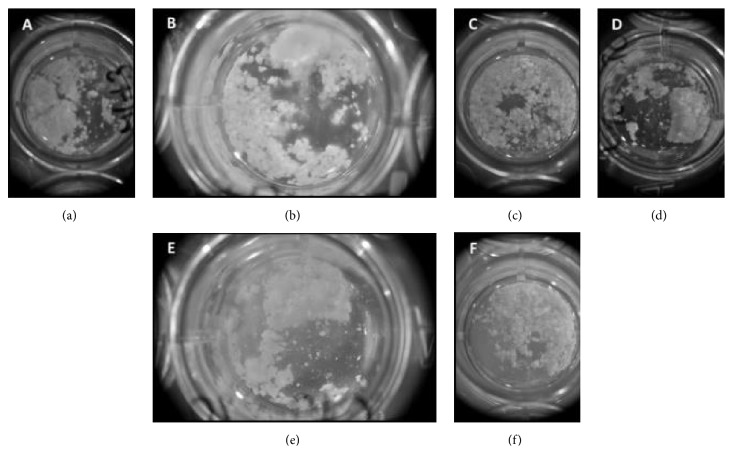
Scaffold morphology after seeding. (a) and (d) 48 hrs after seeding. (b) and (e) 72 hrs after seeding. (c) and (f) 192 hrs after seeding ((a)–(c) 1 mL well volume; (d)–(f) 2 mL well volume).

After 2 hours incubation (37°C at 5% CO_2_), however, all of the scaffolds had begun to leak and significant volumes of solution containing both cellular debris and scaffold debris were clearly visible escaping from the scaffolds. The scaffolds were transferred to 24-well plates and images taken of the leaked solution. Significant segments of scaffold can clearly be seen in Figure 8 with small clumps of cells remaining unattached from the dish surface.

After 24 hrs, it was difficult to distinguish cellular debris from the mass of scaffold debris that had departed from the main scaffold body but nevertheless some cell attachment remained present (Figure 8).

**Figure 9:**
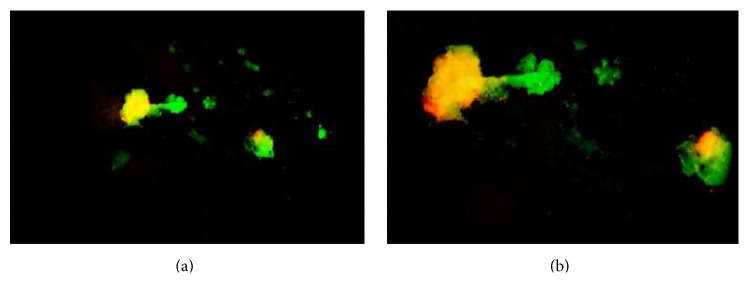
Live/Dead staining carried out on scaffold segments 192 hrs after seeding. (a) 4x magnification. (b) 10x magnification.

Disintegration of the scaffolds continued 48 hrs after seeding. Within 192 hrs (8 days) all of the scaffolds had completely degraded and broken down into small fragments. These fragments are thought to be the TCP component of the scaffolds. Live/Dead staining was carried out on scaffold segments 192 hrs after seeding (Figure 9) and several segments of scaffold showed high cell viability. Fragments were taken from several scaffolds that had completely disintegrated/degraded with each showing a higher than expected cell viability.

During The MTT evaluation, a standard curve producing an *R* ^2^ value of 0.99 with the lowest cell number used for the curve was 0.2 × 10^6^, just 40% of the original seeding density. Within just 45 hrs, cell viability had dropped below this value and continued to drop up to 70 hrs.

## 4. Discussions

In this study, a flexible and biocompatible composite scaffold of collagen and *β*-tricalcium phosphate was prepared without using a chemical cross-linker (via dehydrothermal processing, DHT) and the effects of the process on the mechanical, thermal, and biocompatibility properties of collagen/*β*-TCP scaffolds were examined. For this purpose, a mixture of ceramic and collagen phases was prepared and subjected to freeze-drying, resulting in cross-linked scaffolds.

The duration of DHT processing was found to have a critical influence on the mechanical features of the scaffolds prepared. The results from compression testing revealed that a 3-day processing time resulted in scaffolds with higher mechanical strength. Increasing the time of exposure to 5 days was found to increase the amount of collagen denaturation in the scaffolds. Although the increase of DHT temperature improves cross-link density, the increase in DHT processing time was found to be inversely proportional to cross-link density, coherent with reported literature [16]. However, the compressive modulus of the scaffolds, determined to be between 2.2 and 3.0 MPa, remains significantly higher than those of similar scaffolds reported in literature [16–19].

FTIR analysis results have established that the collagen fibers were cross-linked via DHT. Additionally, the FTIR spectrum indicates that gamma irradiation further increased cross-link density. Bands up to a width of 1200 cm^−1^ belong the PO_4_^−3^ of *β*-TCP [20]. Although the amide-I (1639 cm^−1^), amide-II (1543 cm^−1^), and amide-III (1452 cm^−1^) bands of collagen are quite similar to those of unmodified collagen, the increase in cross-link density due to the interaction between carboxyl and amino groups has led to a shift in the wavenumber and decrease in the area below the peak [21]. Additionally, a cross-link between lysine and alanine may have occurred due to DHT processing; however, this may remain hidden in the FTIR spectra [16].

The denaturation temperature of collagen was found, with differential scanning calorimetry analysis, to be about 50°C. Whilst the denaturation temperature (*T*_*d*_) causes the destruction of the triple-helix structure of collagen, the degradation temperature (*T*_*m*_) relates to the breaking of covalent bonds within the collagen molecule [22]. Several factors including the collagen isolation technique, type of collagen, and water content will affect the denaturation temperature [23]. Whilst DHT processing improves cross-link density, it also simultaneously leads to the denaturation of collagen molecules [24]. Along with this, the moisture content of the scaffold or the accumulation of solvent within it will change the denaturation temperature [16] and to overcome this problem we have utilized a lyophilization procedure prior to cross-linking.

Gamma irradiation (standard dose recommended by the European Pharmacopeia) was used for the sterilization of the collagen/*β*-TCP scaffolds [25]. Although the formation of free radicals is not detected with electrospin resonance (ESR), gamma radiation is known to impede the mechanical properties of the material and speed the degradation process. To overcome this issue a DHT sterilization method, also described in literature [26], could be opted.

The swelling capacity of the scaffolds carries great importance for tissue culture in terms of cell growth and differentiation [27]. Whilst water absorption was seen in scaffolds subjected to 1 day of DHT processing, water absorption levels remained constant for scaffolds, which underwent longer DHT processing times. Swelling capacities depend on collagen and *β*-TCP content as well as cross-link density. A reported study has found a higher swelling ratio, possibly due to the utilization of only collagen matrix [21].

The scaffolds prepared in this study did not cause cytotoxic effects. A Live/Dead staining analysis found a high level of cell viability, implying that collagen/*β*-TCP scaffolds remaining did not negatively affect cell survival. During* in vitro* disintegration tests, *β*-TCP particles disintegrated from the scaffolds. This could be attributed to the excessive cross-linking of the scaffold [28] or long DHT processing times [29]. This disintegration could give increased surface area and vascularization when implanted.

## 5. Conclusion

In this study, we have successfully developed porous collagen/*β*-TCP scaffolds with an interconnected pore structure with *β*-TCP particles homogeneously distributed in the network of collagen fibrils. We have found that these scaffolds display complete biocompatibility, superior mechanical properties and can offer ease of handling. They are able to bend and be rolled, making it suitable for wide range of indications in terms of handling. The optimal DHT time was obtained by using data from characterization studies. Collectively, the findings from this study suggest that these cross-linked collagen/*β*-TCP scaffolds fabricated via dehydrothermal processing show great potential for use in bone tissue engineering applications.

## Figures and Tables

**Table 1:** Thermal properties of collagen/*β*-TCP scaffold.

Groups	Total weight loss (%)	^*∗*^ *T*_*d*_ (°C)	^*∗∗*^ *T*_*m*_ (°C)
Sterile sample	33,3	133,3	46,6
Nonsterile sample	28,6	120,0	53,3

^*∗*^Temperature at degradation. ^*∗∗*^Temperature at denaturation.

**Table 2:** Compression modulus of scaffolds at 1-, 3-, and 5-day DHT processing time (*n* = 3).

Treatment time	1 day	3 days	5 days
Sterile (MPa)	2,32 ± 0,11	3,07 ± 0,15	2,22 ± 0,11
Nonsterile (MPa)	3,67 ± 0,18	3,45 ± 0,17	2,19 ± 0,1

**Table 3:** Scaffold parameters assessed by micro-CT analysis.

Parameter	Amount
Total porosity (%)	65,63
Open porosity (%)	65,54
Surface density (1/mm)	8,84
Pixel size (*µ*m)	13,72
